# First Complete Genome Sequence of a Simian Foamy Virus Isolate from a Cynomolgus Macaque

**DOI:** 10.1128/genomeA.01332-16

**Published:** 2016-12-01

**Authors:** Koji Sakai, Yasushi Ami, Yuriko Suzaki, Tetsuro Matano

**Affiliations:** a AIDS Research Center, National Institute of Infectious Diseases, Tokyo, Japan; b Division of Experimental Animal Research, National Institute of Infectious Diseases, Tokyo, Japan

## Abstract

We report here the first complete proviral genome sequence (DDBJ/ENA/GenBank accession no. LC094267) of a simian foamy virus, SFVmfa/Cy5061, isolated from a cynomolgus macaque (*Macaca fascicularis*). This proviral genome consists of 12,965 nucleotides and has five open reading frames, *gag*, *pol*, *env*, *tas*, and *bet*, as with other foamy viruses.

## GENOME ANNOUNCEMENT

Simian foamy virus (SFV) belongs to the *Spumavirus* genus of the *Spumaretrovirinae* subfamily within the *Retroviridae* family. A wide variety of monkeys and apes are naturally infected with SFVs, but no human foamy virus has been identified (1). SFVs can infect a wide range of animals, including human and its natural host, using heparan sulfate as a receptor (2, 3). So far, no disease association was confirmed both in natural and nonnatural hosts (1). There is a report that preexisting SFV infection could influence simian immunodeficiency virus infection and disease outcome in rhesus macaques (4). Foamy viruses are unique among retroviruses in that the reverse transcription of viral RNA genome occurs during virus production, resulting in the incorporation of proviral DNA in virus particles (1). The proviral genome of SFV is about 13 kbp long and consists of two long terminal repeats (LTRs) and three structural (*gag*, *pol*, and *env*) and two regulatory (*tas* and *bet*) genes. Although many SFV sequences were deposited in DDBJ/ENA/GenBank, no complete proviral SFV genome sequence derived from cynomolgus macaques (*Macaca fascicularis*) has been reported to date.

Here, we report the first complete genome sequence of an SFV isolate derived from a cynomolgus macaque to facilitate the comparative genetic studies of SFVs. We isolated a naturally occurring SFV, SFVmfa/Cy5061, by culture of primary kidney cells obtained from a male cynomolgus macaque from the Philippines at euthanasia. The animal experiment was approved by the Committee on the Ethics of Animal Experiments of the National Institute of Infectious Diseases (NIID) (approval no. 512001) (5) under the guidelines for animal experiments at NIID, in accordance with the Guidelines for Proper Conduct of Animal Experiments established by the Science Council of Japan (http://www.scj.go.jp/ja/info/kohyo/pdf/kohyo-20-k16-2e.pdf). Viral DNA was extracted from the culture supernatant using High Pure viral nucleic acid kit (Roche Diagnostics) (6) and employed for the template for PCR. PCR primer pairs were designed on the basis of the SFV-1 genome sequence (GenBank accession no. NC_010819) to generate a series of 400- to 700-base overlapping PCR products that covered the entire viral genome. DNA fragments amplified by PCR using Premix Taq (TaKaRa Bio) (7) were sequenced by the Sanger method using the fluorescent sequencing system (Applied Biosystems) (8, 9). Sequencing data were compiled and analyzed with the Lasergene software package (DNAStar, Madison, WI, USA). The obtained SFVmfa/Cy5061 proviral genome sequence was 12,965 nucleotides in length and had five open reading frames (*gag*, *pol*, *env*, *tas*, and *bet*) as with other SFV isolates. The nucleotide sequence identities between SFVmfa/Cy5061 and SFV-1 were 85% in whole proviral genome, 84% in LTR, 84% in *gag*, 88% in *pol*, 83% in *env*, 84% in *tas*, and 78% in *bet*. The *bet* gene was found to be more diverse than any other genes. The phylogenetic analysis of the SFVmfa/Cy5061 genome revealed that this virus is clearly distinct from those isolated from rhesus macaques (*Macaca mulatta*).

### Accession number(s).

The complete proviral genome sequence of SFVmfa/Cy5061 has been deposited in DDBJ/ENA/GenBank under the accession number LC094267.
